# Report of a unique case of gemcitabine-induced radiation recall myelitis following spinal cord irradiation

**DOI:** 10.1259/bjrcr.20190118

**Published:** 2020-04-23

**Authors:** Ingrid Masson, Stéphane Supiot, Isabelle Doutriaux-Dumoulin, François Thillays

**Affiliations:** 1 Department of Radiation Oncology, Institut de cancérologie de l’Ouest René-Gauducheau, boulevard Jacques-Monod, 44805 Saint-Herblain, France; 2 Centre de Recherche en Cancéro-Immunologie Nantes/Angers (CRCINA, UMR 892 INSERM), Institut de Recherche en Santé de l'Université de Nantes, Nantes CEDEX 1, France; 3 Department of Radiology, Institut de cancérologie de l’Ouest René-Gauducheau, boulevard Jacques-Monod, 44805 Saint-Herblain, France

## Abstract

Radiation recall is a rare phenomenon, defined as an acute inflammatory reaction in a previously irradiated area, after administration of anti-tumor agents, including chemotherapy. It is most commonly reported to trigger skin reactions but internal organ involvement is possible, particularly with gemcitabine. We report here a unique case of a gemcitabine-induced radiation recall myelitis following spinal irradiation.

A 53-year-old patient received analgesic irradiation of the seventh thoracic vertebra (T7) in the context of metastatic non-small cell lung cancer, at conventional radiotherapy dose and fractionation. She was subsequently treated with gemcitabine and developed myelitis whose chronology is compatible with a radiation recall reaction. Spinal MRI confirmed a T6–T7 spinal cord enhancement, with an associated spinal cord oedema. Corticosteroids and supportive care did not improve myelitis symptoms. The patient died within a year of the radiation recall, due to a metastatic progression of lung cancer.

This is, to our knowledge, the first reported case of gemcitabine-induced radiation recall myelitis and only the third case involving the spinal cord. Radiation recall is a rare and poorly understood phenomenon and all cases should be reported.

## Background

Radiation recall reaction is defined as an acute inflammatory reaction confined to previously irradiated areas that can be triggered when chemotherapy is administrated after radiotherapy.^1^ The aetiology, the incidence and the time before occurrence remain not well known. Radiation recall has been described with a range of chemotherapy agents,^1^ such as taxanes, alkylating agents, anthracyclines, antimetabolites and nucleoside analogues to which gemcitabine belongs. Targeted therapies^2^ and even non-related cancer treatments^1,3^ like antibiotics or statins are described as radiation recall inducing agents too. Its incidence is not possible to establish because of its main description through case reports.

We present here the case of a patient who presented a gemcitabine-induced radiation recall myelitis following palliative spinal cord irradiation.

## Clinical presentation

**Figure 1.**
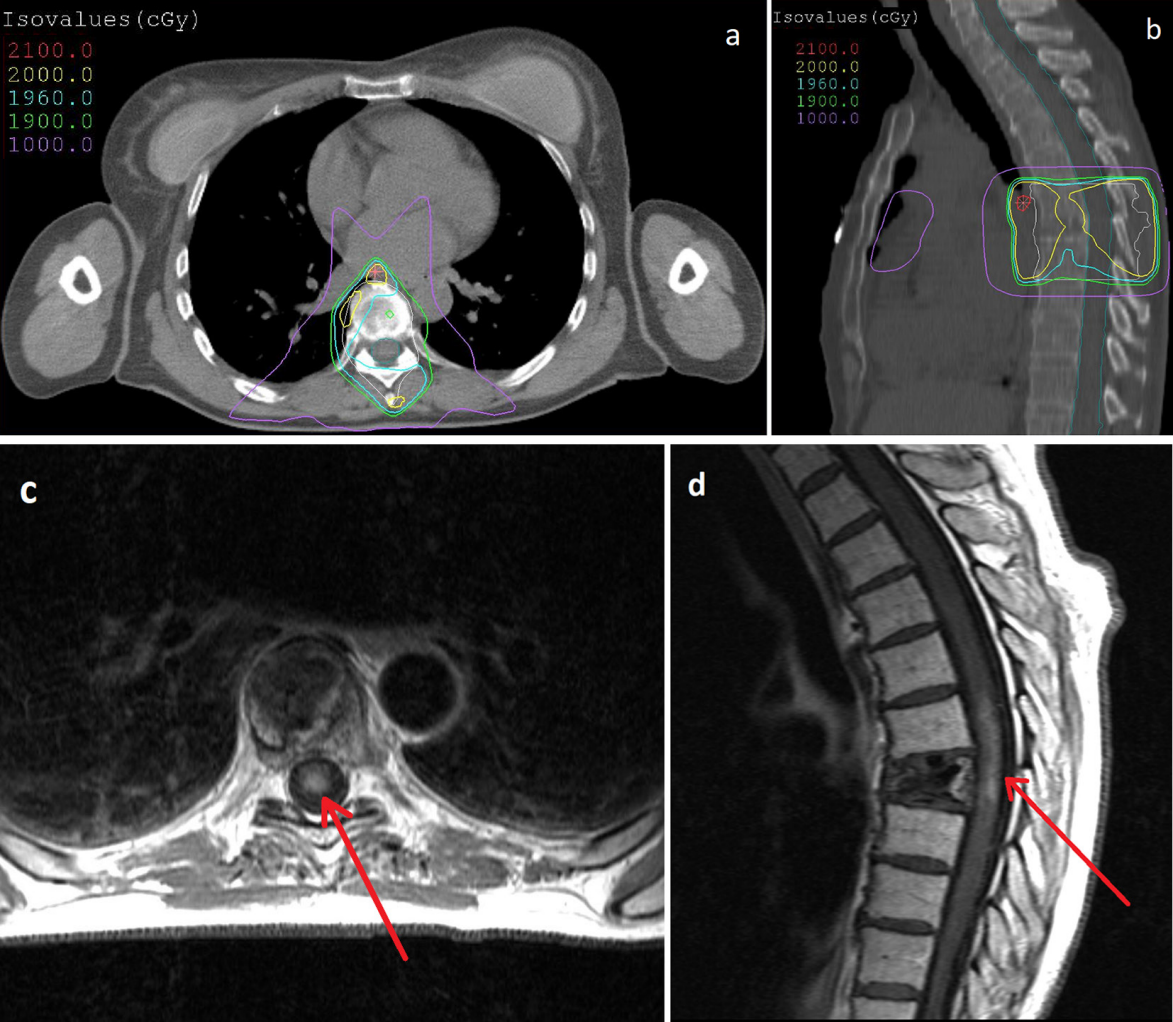
a and b: Treatment planning system (TPS) for T6–T8 irradiation. The balistic (a) was made of 2 right and left posterior oblique beams and one anterior beam. Isovalues lines in axial (a) and sagittal (b) plan showed a homogeneous coverage of the planning target volume (PTV). “Hot spot” (21 Gy) was localised forward rachis (b). Figure 1.c and d: Myelitis on the spinal MRI (T1 SE FAT SAT GADO sequence). After injection of gadolinium chelate, a 36 mm intraspinal contrast enhancement appeared in T6–T7, representing myelitis (d, red arrow). In axial section centred on T6–T7 myelitis predominated in the posterior part of spinal cord (c, red arrow). Cement was visible in T7 vertebral body.

**Figure 2.**
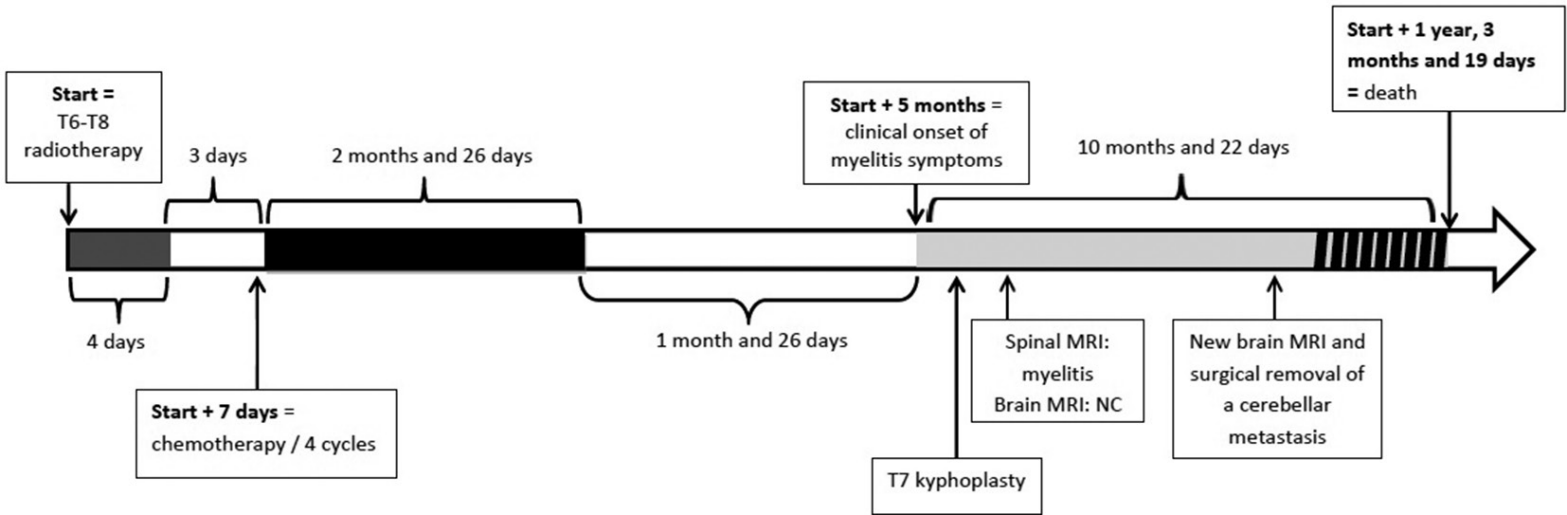
Timeline of events NC: non-conclusive. The section with the striped black lines shows the metastatic progression of cancer.

A 53-year-old female, with no significant medical history, was diagnosed with painful lytic bone lesion of T7 vertebra. Her only treatment was opioids, recently introduced for the treatment of spinal pain. There were no complications such as posterior wall damage or epiduritis. The patient had no neurological symptom. This bone lesion led to the diagnosis of non-small cell lung cancer with a bone metastasis in T7 vertebral body. The patient was first treated with radiotherapy to T6–T8 vertebrae, for pain relief. Twenty Gray (Gy) were delivered in four fractions of 5 Gy and in 4 days, by a three-dimension conformal radiation therapy technique (Figure 1a. and b.). A Grade 2 oesophagitis was the only acute toxicity following irradiation, according to Common Terminology Criteria for Adverse Events (CTCAE) classification. Three days later, the patient started chemotherapy with cisplatin and gemcitabine (1250 mg/m²) for a total of four cycles spread over the course of 2 months and 26 days. No other systemic treatments (including chemotherapy, targeted therapy and immunotherapy) were used. The patient had a partial response to chemotherapy with no unusual toxicity. Approximately 5 months after the completion of radiotherapy (147 days) and 1.5 months after the completion of chemotherapy (57 days), the patient had reported the onset of parenthesis affecting the lower extremities and the persistence of intense, opiate-resistant T7 spinal pain. A T7 kyphoplasty was programmed. This surgical procedure went well with a rapid analgesic effect and no complication. However, parenthesis gradually increased, on a bilateral basis, until T7 dermatome and were associated with sphincter disorders. Other neurological symptoms appeared, corresponding to an acute posterior cord syndrome with a Lhermitte's phenomenon and proprioceptive dysfunction (squeezing feeling). A timeline of events is presented in Figure 2.

### Imaging findings

A spinal MRI was performed, because of the strong suspicion of epiduritis or spinal cord compression. Imagery found a known T7 vertebral collapse, associated with post-kyphoplasty rearrangements, without posterior wall defect or cement extravasation. On *T*_1_-weighted images, there was an enlarged thoracic spinal cord. After injection of gadolinium chelate (Figure 1c. and d.), an intraspinal contrast enhancement was localised in T6–T7 (1d) and predominating on the posterior part of the spinal cord on T6–T7 axial MRI images (1c). These images were in favour of myelitis. *T*_2_-weighted images showed a high signal intensity super-jacent and subjacent to the contrast enhancement, corresponding to a spinal cord oedema. There was no other abnormality. A brain MRI did not find any prove of brain metastasis or carcinomatous meningitis.

### Follow-up

Myelitis symptoms did not improve, despite various treatments: corticosteroids (prednisolone 20 mg daily), opioids (oxycodone 20 mg in the morning and 30 mg at night), anticoagulants (Tinzaparin) or neuropathic pain treatments (pregabalin 200 mg daily). A paraparesia and brisk bilateral patellar tendon reflexes installed progressively. Regarding the disease evolution, the T7 vertebral metastasis was controlled by radiotherapy. A single cerebellar metastasis appeared in the follow-up and was surgically removed. The patient died within a year of the radiation recall, due to a metastatic progression of lung cancer. This myelitis was unexpected. The spinal irradiation delivered a low dose, with conventional fractionation and without unusual acute toxicity. The kyphoplasty could not explain the symptoms in view of the prior symptoms and later worsening, at distance from the interventional radiology procedure. The only plausible explanation is a radiation recall effect on spinal cord, triggered by gemcitabine, a nucleoside analogue.

## Discussion

Radiation recall generally occurs from 8 days to years after the irradiation.^1,2,4^ It must be distinguished from radio-sensitisation reaction, which occurs within a week of radiation exposure. The radiation recall myelitis must be also distinguished from a case of radiation myelopathy. In our case report, the irradiation at doses far below the 45 Gy threshold with conventional fractionation, the fast onset of neurological symptoms, the lack of recovery after a few months do not support the hypothesis for either early delayed or late injury of radiation myelopathy as described by Wong et al.^5^

The underlying pathophysiological mechanism of radiation recall remains unclear. Several hypothesis have been proposed: depletion or modifications in function or increased sensitivity of stem cells within the irradiated field, vascular damages.^4^ The current hypothesis is a drug hypersensitivity reaction: some systemic agents may be able to trigger a non-immune inflammatory reaction in patients whose inflammatory response threshold has been reduced by irradiation.^4,6^

Theoretically, all organs may be concerned by radiation recall effect: many descriptions involve skin.^4,7,8^ Some cases have been described in musculoskeletal system, lungs, head and neck, gastrointestinal tract and lymphatic system.^2,9,10^ There are fewer cases of radiation recall in the central nervous system (CNS) and they were mostly reported with gemcitabine. One case of optic neuritis was reported after whole-brain radiotherapy to a total dose of 40 Gy and subsequent treatment with eight cycles of gemcitabine 1000 mg/m2.^9^ One case of brainstem necrosis was described, following radiotherapy of a metastasis to clivus to a total dose of 35 Gy and eight cycles of gemcitabine 600 mg/m^2^.^9^ In addition to our case report, two cases of radiation recall myelitis have been described, but with paclitaxel chemotherapy in breast cancer^11^ or dabrafenib, a BRAF inhibitor in metastatic melanoma.^12^ The first one occurred 8 months after stereotactic body radiotherapy (SBRT) to the T3–T5 vertebral bodies, to a dose of 30 Gy in 6 Gy/fraction and during paclitaxel chemotherapy.^11^ The second case was a T2–T9 myelitis that occurred 8 months after SBRT for lung metastasis and triggered by dabrafenib.^12^ To our knowledge, our case is the first reported case of radiation recall myelitis described with gemcitabine. One of the theories proposed by Jeter et al to explain the greater capacity of gemcitabine, compared to other drugs, to induce recall effects in the CNS is a higher potential capability to cross-the blood–brain barrier.^9^ It must be considered carefully because of the morphological and functional differences between blood–spinal cord barrier and blood–brain barrier and the effect of radiotherapy on capillary endothelium permeability.^13^ Gemcitabine may be more likely to cause unusual radiation recall reactions, with organ involvement,^14^ compared to other drugs that mostly trigger dermatitis or mucositis..^9^ Gemcitabine is a potent radiation enhancer whose mechanism is not fully understood. It induces a dose-dependent inhibition of DNA synthesis and an induction of cells apoptosis in S phase.^15^ This may also partly explain the involvement of organs such as the CNS in the gemcitabine-induced radiation recalls. Cisplatin may contribute to the reaction^16^ but cannot, on its own, explain the myelitis, given the small number of cases reported with platinum salts.^1,6,9^ In our case, myelitis appeared with conventional fractionation in palliative radiotherapy to a dose of 20 Gy in four fractions, unlike the two cases of myelitis previously described, which were after SBRT.

Most authors report a response to supportive therapy under the condition of the withdrawal of the causal agent.^1,6^ This was not possible for our patient, for whom gemcitabine was no longer administered at the time of the myelitis. Responses to radiation recall with corticosteroids, non-steroidal anti-inflammatory drugs,^1^ hyperbaric oxygen or surgery have been previously described.^6,9^ However, our patient’s symptoms did not improve with corticosteroid or supportive care. This is consistent with most of the other cases of radiation recall involving the CNS.^9,11^ It is not possible to avoid radiation recall but risk can be decreased by prolonging the interval between the completion of radiotherapy and the initiation of chemotherapy.^1,6^ The radiation recall seems to be indeed most severe with a shorter time interval.^6,14^ In our case report, this delay is only 3 days, which may at least partially explain the severity of the myelitis and the lack of improvement despite all the supportive care introduced.

## Conclusion

To our knowledge, this is the first reported case of a gemcitabine-induced radiation recall myelitis. Any case of radiation recall should be collected, in order to improve our knowledge of this rare and unclear phenomenon.

## Learning points

- Radiation recall is a rare and still unexplained phenomenon.
- This diagnosis should be considered in the presence of unusual toxicity occurring after irradiation and in the context of triggering factors (chemotherapy, targeted therapy and medication).
- This case is the first reported case of gemcitabine-induced radiation recall myelitis following spinal cord irradiation. All cases should be reported and collected to improve understanding of the radiation recall mechanism.
